# Real-Time Georeferencing of Fire Front Aerial Images Using Iterative Ray-Tracing and the Bearings-Range Extended Kalman Filter

**DOI:** 10.3390/s22031150

**Published:** 2022-02-02

**Authors:** Bernardo Santana, El Khalil Cherif, Alexandre Bernardino, Ricardo Ribeiro

**Affiliations:** 1 Instituto Superior Técnico, Av. Rovisco Pais 1, 1049-001 Lisboa, Portugal; bernardo.santana@tecnico.ulisboa.pt; 2 Institute for Systems and Robotics, Instituto Superior Técnico, Av. Rovisco Pais 1, 1049-001 Lisboa, Portugal; c.elkhalil@uae.ac.ma (E.K.C.); ribeiro@isr.tecnico.ulisboa.pt (R.R.)

**Keywords:** forest fire, aerial vehicle, georeferencing, iterative ray-tracing, cubature kalman and bearings-range filters, GPS, IMU, DEM

## Abstract

Although Aerial Vehicle images are a viable tool for observing large-scale patterns of fires and their impacts, its application is limited by the complex optical georeferencing procedure due to the lack of distinctive visual features in forest environments. For this reason, an exploratory study on rough and flat terrains was conducted to use and validate the Iterative Ray-Tracing method in combination with a Bearings-Range Extended Kalman Filter as a real-time forest fire georeferencing and filtering algorithm on images captured by an aerial vehicle. The Iterative Ray-Tracing method requires a vehicle equipped with a Global Positioning System (GPS), an Inertial Measurement Unit (IMU), a calibrated camera, and a Digital Elevation Map (DEM). The proposed method receives the real-time input of the GPS, IMU, and the image coordinates of the pixels to georeference (computed by a companion algorithm of fire front detection) and outputs the geographical coordinates corresponding to those pixels. The Unscented Transform B is proposed to characterize the Iterative Ray-Tracing uncertainty. A Bearings-Range filter measurement model is introduced in a sequential filtering architecture to reduce the noise in the measurements, assuming static targets. A performance comparison is done between the Bearings-Only and the Bearings-Range observation models, and between the Extended and Cubature Kalman Filters. In simulation studies with ground truth, without filtering we obtained a georeferencing Root Mean Squared Errors (RMSE) of 30.7 and 43.4 m for the rough and flat terrains respectively, while filtering with the proposed Bearings-Range Extended Kalman Filter showed the best results by reducing the previous RMSE to 11.7 and 19.8 m, respectively. In addition, the comparison of both filter algorithms showed a good performance of Bearings-Range filter which was slightly faster. Indeed, these experiments based on the real data conducted to results demonstrated the applicability of the proposed methodology for the real-time georeferencing forest fires.

## 1. Introduction

Forest fires represent one of the biggest catastrophes affecting the land [1]. Every year, over 3400 km² of land were burned in the European Union [2]. A great percentage of this burned area includes protected ecosystem areas which will take many years to restore [2]. The problem is exacerbated due to the difficulties of identifying, managing and predicting the fire propagation [1,3,4]. On 17 June 2017, a complex of at least five wildfires converged in the region of Pedrógão Grande, Portugal, and burned more than 450 km² while taking the life of 66 persons [5]. After that dramatic event, a large national research funding program for wildfire combat and prevention took place. On that context, the FIREFRONT project (www.firefront.pt, accessed on 15 September 2021) is an initiative to help combat wildfires using automated image analysis and georeferencing techniques from airborne imagery for real-time decision support. The ability to detect, map and forecast the progression of fires is essential to adequately plan its combat strategy. In this paper, we describe a study on the techniques to compute the geographical location of the coordinates of observed image pixels corresponding to a fire front. The methods for detecting the fire front pixels in images are outside the scope of the present paper, and have been described in [6,7].

In recent years, remote sensing has seen an increased interest in the scientific community [8,9,10,11]. The development and widespread of Unmanned Aerial Vehicles (UAVs), as a cheaper solution when compared to manned aerial vehicles, enabled the development of target geolocalization applications. These range from agriculture [12], natural disaster management [13] and fire detection and monitoring [14].

The most commonly used sensors regarding target geolocalization are digital cameras, Global Positioning Systems (GPSs) and Inertial Measurement Units (IMUs). This became known as Direct Georeferencing, since direct sensor orientation is computed by processing the information provided by the onboard sensors [15], i.e., the camera’s extrinsic parameters (EPs) are directly calculated. Depending on the accuracy requirements, sensors with different specifications can be used. However, the higher the desired accuracy, the higher cost and/or size of the hardware. Due to payload constraints, UAV’s typically use smaller and error-prone IMU’s, especially in yaw measurements [16], such as MicroElectroMechanical Systems (MEMS). This lack of quality lead to the development of computer vision algorithms such as Structure from Motion (SfM) that extract and match features between images. The integration of these algorithms with the poses provided by the IMU and GPS greatly increase the accuracy of the georeferencing process.

Alternatively, Indirect Georeferencing can be used for systems that lack navigation equipment. This requires, however, the placement of Ground Control Points (GCP) to determine the camera’s EPs, which can be time consuming and costly [17]. Furthermore, in a natural disaster scenario or in rough and inaccessible terrains, it is impracticable to place GCP. Thus, our work focus on the use of Direct Georeferencing methods to enable real-time wildfire geolocalization.

There are several studies on direct georeferencing methods that motivate our approach. Sheng [18] and Sheng [19] test three different algorithms to solve the optic ray-DEM intersection: Iterative Photogrammetry (IPG), Ray-Tracing (RT) and Iterative Ray-Tracing (IRT). Ponda et al. [20] developed a Line-of-Sight Bearings-Only EKF for target localization to filter the noise in the raw observations of the line-of-sight. Xu et al. [21] proposed to use the Cubature Kalman Filter (CKF) on the same measurement model (Bearings-Only) to take into account the possible linearization errors induced by the standard EKF. Zhang et al. [22] proposed a Bearings-Only model with a CKF for relative spacecraft attitude and orientation estimation. Later, Zhang et al. [23] presents a target geolocation method for UAV’s where range measurements are obtained from stereo vision but did not use a filtering.

In this work we propose a georeferencing algorithm for forest environments composed of a EKF with Bearings-Range observation model, where the range is computed via IRT. This combination is novel as previous filtering architectures use Bearings-Only observations. Although Bearings-Range Extended Kalman Filters are common in robotics [24], to the best of the authors knowledge, they have not been applied to target georeferencing with measurements obtained by Iterative Ray-Tracing. Additionally, we propose using Unscented Transform (UT) to characterize the uncertainty of the bearing and range observations in the initialization of the filter. This uncertainty can also be useful in later stages of the project, e.g., in making more realistic stochastic simulation of fire propagation. We perform experiments both in simulations and real imagery. In the simulations we perform a comparative study between the unfiltered IRT measurements and filtered versions of it with EKF/CKF filters and Bearings-Only/Bearings-Range observation models. We compare the target geolocalization uncertainties in flat vs. hilly terrains. In real imagery we test the algorithm with footage from real fire combat situations and from smartphone data, assessing the quality of the estimations according to different viewpoints. Finally, we perform an experiment with an indirect method using manually selected GCP to define a benchmark and qualitatively compare performance with the direct methods. However, we note that indirect methods are not easy to automate and not suitable to our problem due to scarcity of GCP in general forest environments.

The paper is organized as follows. In Section 2 we review the main approaches for georeferencing targets observed on images acquired from aerial vehicles. In Section 3 we present the outline of the proposed algorithms and the methods used in its implementation. In Section 4 we present simulation results that allow the quantitative assessment of the performance of the proposed methods and a comparison between different algorithmic settings and environmental conditions. In Section 5 we present results with real images and videos, including actual firefighting scenarios, and present a discussion of the obtained results. Finally, in Section 6 we summarize the main conclusions of the paper and discuss some open issues for future work.

## 2. Georeferencing Methods for UAV Observations

This section outlines the problem of target georeferencing from airborne cameras and provides a summary of the main approaches proposed in the literature.

### 2.1. Optic-Ray Surface Intersection

When projecting a 3D point from the world to the 2D image plane, there is a loss of information. The 2D points are defined up to an unknown scale factor, defined by the distance from the 3D point to the principal point of the camera. To invert the projection, assuming that the camera’s intrinsic parameters (IP) and extrinsic parameters (EP) are known, either the distance to the observed point or information regarding the surface where the point lies needs to be known.

### 2.2. Flat Earth Hypothesis

Leira et al. [25] propose the intersection of the optic ray, whose direction is defined by the pixel to map, with a plane with altitude Z equal to zero. The UAV is equipped with a gimbal and a thermal camera, and the transformation between the camera frame and the inertial frame is known. By doing this assumption, the scaling factor can be calculated, and the optic ray is projected to the surface. An accuracy of 7.8 m was achieved for a variable flight height, between 50 and 100 m. Xiang and Tian [26] propose an automatic georeferencing algorithm that estimates the target’s world coordinates and mosaics the images together based on their estimated geographical positions. Since the purpose of that paper was to find horizontal coordinates, it assumes that all targets are on the same elevation plane.

### 2.3. Digital Surface

Sheng [18] tests three different algorithms to solve the optic ray-DEM intersection: Iterative Photogrammetry (IPG), Ray-Tracing (RT) and Iterative Ray-Tracing (IRT). The IPG’s convergence depends on the initial elevation, the view angle and profile inclination angle and is prone to fail with occlusions or rough terrains while IRT’s convergence depends on the step chosen to iterate along the optic ray. RT actually calculates the intersection point, so it is the most robust method; however, it is more computationally demanding when compared to IRT and IPG. If computational power is available, the best option is the RT, otherwise, the IRT seems the best option as it is not so prone to fail with occlusions as the IPG and its convergence only depends on the step size.

Sheng [19] continues the previous work and reviews in detail the IPG, as it is the most promising and efficient of the methods. The convergence condition and convergence speed are analyzed.

### 2.4. Structure from Motion

Structure from Motion is a method that relies on feature extraction and matching from sequential images captured with different camera poses. Having multiple images with the 2D coordinates of these features enables stereo-vision techniques to solve their 3D coordinates. These coordinates are then georeferenced in two possible ways: the camera pose in the inertial frame is known, via GPS and IMU, or with the placement of GCP. SfM is usually followed by a Bundle Adjustment (BA), an algorithm that takes as input the targets’ 3D and 2D positions in the camera and image frames, respectively, and performs a least-squares minimization with the reprojection error as a cost function. Forlani et al. [27] propose the use of SfM to match a set of images acquired under poor Global Navigation Satellite System (GNSS) coverage, designated as the master block, to a set of georeferenced images acquired under nominal GNSS coverage, designated as the auxiliary block. Features are extracted and used to match the images from the master to the auxiliary blocks and are followed by a BA. The method was tested with different camera calibrations. A Root Mean Squared Error (RMSE) of centimeters was achieved for a master block flown at 30 m and an auxiliary block flown at 100 m. He et al. [17] present the mathematical premise of SfM with detail. Prior knowledge of the vehicle trajectory (planar motion) is used to simplify the problem from 6 Degrees Of Freedom (DOF) to 3DOF, enabling a 2-point approach. An incremental approach is compared to a global approach in terms of EP recovery, followed by a BA. The RMSEs obtained for both methodologies are on the centimeter order, with the global strategy performing slightly better than the incremental, for a maximum flight height of 50 m.

### 2.5. Georeferenced Imagery

This methodology consists of matching images captured by an aerial vehicle with available georeferenced imagery, such as Google Earth. Conte et al. [28] propose an image registration approach by pattern-matching the images collected at 100 m from a micro aerial vehicle with satellite imagery. Multiple measurements are taken and a recursive least square filter is applied. The method is compared against the intersection of the optic ray assuming the flat earth simplification. The proposed method achieved a RMSE of 2.25 m while the intersection method best result was a RMSE of 22 m. Hamidi and Samadzadegan [29] propose the IPG algorithm combined with EP refinement using feature matching with georeferenced imagery. The DEM used was the Shuttle Radar Topography Mission [30], with a spatial resolution of 90 m. On a first stage, the EP are computed using the information provided by the UAV’s IMU and GPS. On a second stage, the EP are adjusted and the IPG is applied. The mean UAV height in the experimental procedure was 400 m. The refinement improved the position accuracy by 100 m to 14.476 m.

### 2.6. Line-of-Sight Filtering

Barber et al. [31] applies the flat earth simplification to calculate the target’s coordinates, but its main innovation is applying a recursive least square filter to multiple observations while performing a loitering pattern. By doing so, the geolocation errors decreased from 40 m to less than 5 m. Ponda et al. [20] introduces a bearings-only 3D target coordinate estimation with an EKF. This model filters the azimuth and elevation angles between the aerial vehicle and the target for every measurement. Three cases are simulated: stationary target, slow-moving target and fast-moving target. In all cases, a stationary process is assumed and the process noise is tuned to allow unknown target motion. The filter manages to track the target for the stationary and slow-moving cases and fails to do so in the fast-moving case. Xu et al. [21] also estimate the target’s position by filtering multiple bearing measurements with a CKF [32] while performing a loitering trajectory, centered on the target. The filter’s initial state is calculated with the IPG using the ASTER-GDEM V2 [33], which has 30-m spatial resolution. The CKF method is compared against the standalone IPG and flat earth simplification. Two experiments are performed on different terrains, rough and flat. In the two cases, the standalone IPG achieves similar accuracy, 39.6 and 36.2 m. In the flat terrain, the CKF and the flat earth simplification achieve similar RMSEs, 10.8 and 12.9 m, respectively. Finally, in the rough terrain, the flat earth has a bad performance (105.6 m), because this approximation is invalid for this terrain type, whereas the proposed method achieves a RMSE of 13.8 m. For both experiments, the UAV flew at a maximum height of 500. Finally, Shao et al. [34] proposed a new model, which can appropriately represent the actual field of view (FOV) for a camera as a filtering step used for positioning data and other sensor measurements.

### 2.7. Others

Zhang et al. [23] use a stereo-vision technique to determine the target relative height with respect to the UAV. In addition, the sensor technology used in UAVs is very similar to that used in today’s smartphones [35]. A yaw bias estimation algorithm is also proposed. Two different trajectories were tested, overflight and loitering. First, the relative target height and yaw bias are estimated using multiple bearing measurements of the same target. Then its coordinates are estimated. The proposed method achieves a horizontal and vertical accuracy of 0.7 and 0.5 m, respectively, for the loitering trajectory, and 1.77 and 1.15 m for the overflight, for maximum flight heights of 20 m. That work provides an interesting result by showing that the trajectory of the UAV is an important factor to take into account when determining the target’s position. Zhang et al. [36] continue the work developed in [23] and study the influence of the trajectory on the accuracy of the georeferencing algorithm. Trajectory planning was again demonstrated to influence the georeferencing accuracy.

## 3. Methodology

In this section, we present the methods used in the proposed georeferencing algorithm. We start with a description of the geometry of the problem with corresponding coordinate frames and, then, we describe the Iterative Ray-Tracing method used, the Unscented Transform, and the Bearings-Range measurement model. Finally, we describe the metrics used in the evaluation. Our method assumes that the camera and IMU Bias are calibrated *a priori*.

### 3.1. Camera Calibration

The camera calibration is an important procedure in computer vision that calculates the camera’s intrinsic parameters and allows the extraction of metric information from bi-dimensional images. For this reason, before taking off, the camera must be calibrated. If the lens configuration is not manipulated, these parameters remain constant throughout the flight.

Using one of the many computer vision tools available (OpenCV or MATLAB Image Processing and Computer Vision Toolbox), the calibration is done by moving and changing the pose of a known pattern in the camera’s line of sight [37]. Usually, this pattern is a checkerboard, and the size of the checkerboard square is measured beforehand.

The output of the calibration process is the camera’s intrinsic parameters matrix $K_{\mathrm{int}}$:

$$K_{\mathrm{int}}=\left[\begin{array}{ccc} f_{x} & 0 & c_{x}\\ 0 & f_{y} & c_{y}\\ 0 & 0 & 1 \end{array}\right],$$

where $\left(f_{x},\;f_{y}\right)$ represent the focal length and $\left(c_{x},\;c_{y}\right)$ is the camera’s principal point. These parameters are essential to define the observation directions through the optic ray from the projection center to the target pixel.

### 3.2. Coordinate Frames

**Figure 1:**
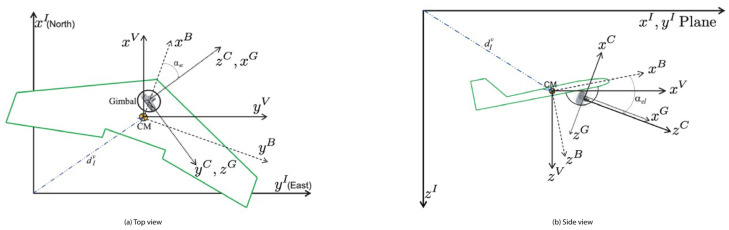
UAV, gimbal and camera frames [31].

The geometry or our problem is illustrated in Figure 1. The camera is installed in a gimbal system that rotates in azimuth and elevation angles to control the observation direction. The gimbal system base and the IMU are rigidly attached to the body of the aircraft. Five coordinate frames were considered: camera, gimbal, body, vehicle and inertial (world) frames. These frames are denoted respectively by: $F^{C}=\left(x^{C},y^{C},z^{C}\right)$, $F^{G}=\left(x^{G},y^{G},z^{G}\right)$, $F^{B}=\left(x^{B},y^{B},z^{B}\right)$, $F^{V}=\left(x^{V},y^{V},z^{V}\right)$ and $F^{I}=\left(x^{I},y^{I},z^{I}\right)$. The vehicle’s frame is a North-East-Down frame centered on the vehicle’s center of mass, where the GPS and IMU are supposed to be located, and has the same orientation as the inertial frame. In fact, since the translations between the camera, gimbal, body and vehicle are typically much smaller than the distances to targets, we can neglect them and simply calibrate the rotation transformations between those frames. Therefore, for the sake of simplicity, we only use the rotation transformations between those frames in the following calculations.

#### 3.2.1. Camera Frame

The camera frame has its origin in the optical center, the $x^{C}$ axis points to the right of the image plane, the $y^{C}$ axis points downward on the image plane and $z^{C}$ axis points in the direction of the optical axis of the camera. The rotation from the camera to the gimbal coordinate frame is, by construction, a simple permutation of the coordinate axes, defined by:

$$R_{C}^{G}=\left[\begin{array}{ccc} 0 & 0 & 1\\ 1 & 0 & 0\\ 0 & 1 & 0 \end{array}\right]$$

#### 3.2.2. Gimbal Frame

The gimbal coordinate frame has two degrees of freedom around $y^{G}$ and $z^{G}$ due to its pan and tilt movements, respectively. Defining the pan (elevation) and tilt (azimuth) angles as $\alpha_{el}$ and $\alpha_{az}$, the rotation from the gimbal coordinate frame to the body coordinate frame is given by $R_{G}^{B}=R_{z}\left(-\alpha_{az}\right)R_{y}\left(-\alpha_{el}\right)$, resulting in:

$$R_{G}^{B}=\left[\begin{array}{ccc} c\alpha_{el}c\alpha_{az} & -s\alpha_{az} & s\alpha_{el}c\alpha_{az}\\ s\alpha_{az}c\alpha_{el} & c\alpha_{az} & s\alpha_{az}s\alpha_{el}\\ -s\alpha_{el} & 0 & c\alpha_{el} \end{array}\right]$$

where cλ≜cosλ and sλ≜sinλ.

#### 3.2.3. Body Frame

The body frame describes the aircraft Bpose (position and orientation) and has its origin in the center of mass of the UAV. The $x^{B}$ axis points in the direction of the nose, the $y^{B}$ axis points towards the right wing and the $z^{B}$ axis points towards the aircraft’s belly. Defining the roll (ϕ), pitch (θ) and yaw (ψ) angles as the movement of the UAV around the axis $x^{B}$, $y^{B}$ and $z^{B}$, respectively, the rotation from the body to the vehicle coordinate frame is defined by $R_{B}^{V}=R_{z}\left(-\psi \right)R_{y}\left(-\theta \right)R_{x}\left(-\phi \right)$, i.e.,

$$R_{B}^{V}=\left[\begin{array}{ccc} c\psi c\theta & c\psi s\theta s\phi -s\psi c\phi & c\psi s\theta c\phi +s\psi s\phi \\ s\psi c\theta & s\psi s\theta s\phi +c\psi c\phi & s\psi s\theta c\phi -c\psi s\phi \\ -s\theta & c\theta s\phi & c\beta c\phi \end{array}\right]$$

### 3.3. Iterative Ray-Tracing

Iterative Ray-Tracing is a methodology that efficiently computes in an approximate intersection between the optic ray corresponding to a camera pixel and the DEM of the observed area. In our work we use the EU DEM v.1.1 [38] which has an approximate resolution of 20m on the locations that will be used in the experimental studies.

Defining ${\vec{P}}_{Target}^{V}$ as a vector, of arbitrary scale, pointing from the UAV to the target in the inertial coordinate frame and ${\vec{P}}_{V}^{I}$ as the vector pointing from the inertial frame origin to the UAV, the target position will be found along the ray starting at ${\vec{P}}_{V}^{I}$ with the direction of ${\vec{P}}_{Target}^{V}$. To obtain ${\vec{P}}_{Target}^{V}$ (Equation (1)), the vector pointing from the camera optical center to the target pixel (u,v) must be defined. Assuming $f_{x}\approx f_{y}$ we obtain:

$${\vec{P}}_{Target}^{C}={\left[\begin{array}{c} u-c_{x},\;v-c_{y},\;\frac{f_{x}+f_{y}}{2} \end{array}\right]}^{T}$$

This vector is now transformed to the vehicle coordinate frame using the coordinate frame transformations described in Section 3.2,

(1) $${\vec{P}}_{Target}^{V}={\left[X_{d}^{\prime }\;Y_{d}^{\prime }\;Z_{d}^{\prime }\right]}^{T}=R_{B}^{V}R_{G}^{B}R_{C}^{G}{\vec{P}}_{Target}^{C}$$

Given the camera position $R_{0}={\left[X_{s}\;Y_{s}\;Z_{s}\right]}^{T}$ in the inertial frame and the normalized pointing vector $R_{d}$ from the aerial vehicle to the target, in the vehicle frame is given by:

$$R_{d}={\left[X_{d}\;Y_{d}\;Z_{d}\right]}^{T}=\frac{1}{\left|\right|{\vec{P}}_{Target}^{V}\left|\right|}\;\cdot \;{\vec{P}}_{Target}^{V}$$

the ray R that starts in the vehicle and points to the target in the inertial frame is parametrically defined

(2) $$R\left(t\right)=R_{0}+t\;\cdot \;R_{d}=\left[\begin{array}{c} X_{s}\\ Y_{s}\\ Z_{s} \end{array}\right]+t\;\cdot \;\left[\begin{array}{c} X_{d}\\ Y_{d}\\ Z_{d} \end{array}\right]$$

where *t* is the step and represents the distance between a point R(t) on the ray and the origin $R_{0}$. When the ray elevation $Z_{R}$ becomes less than the surface elevation $Z_{\mathrm{DEM}}$, the intersection is detected. To do this, we use a method with a dynamic step size for *t*. The algorithm is initialized with a large step value *t* and when the intersection with the DEM is detected ($Z_{R}$ ≤ $Z_{\mathrm{DEM}}$), the step size is reduced with a step divider, $t_{\mathrm{div}}$, until the *t* becomes smaller than a pre-defined threshold, $t_{\mathrm{th}}$ (Algorithm 1). Furthermore, the starting iteration point, $R_{0}^{\prime }$ (Equation (3)), is set as the intersection of the ray with the maximum elevation of the loaded DEM with respect to the inertial frame, $Z_{\max}$. By defining a scaling factor

$$\lambda =\frac{Z_{\max}-Z_{R_{0}}}{Z_{d}},\;\mathrm{for}\;\lambda >0$$

where $Z_{R_{0}}$ is the height of the camera and $Z_{d}$ is the third component of the normalized pointing vector, it is possible to calculate the new starting point, with $Z_{R_{0}}$ = $Z_{\max}$

(3) $$R_{0}^{\prime }=R_{0}+\lambda \;\cdot \;R_{d}$$

Finally, bilinear interpolation was implemented, as in [21], to refine the elevation of the queried point. Ghandehari et al. [39] concluded in their work that for DEM’s with finer resolutions, such has the EU-DEM v1.1 [38], the one used in this work, this type of interpolation achieves good results with low processing times.
**Algorithm 1:** Complete Iterative Ray-Tracing.
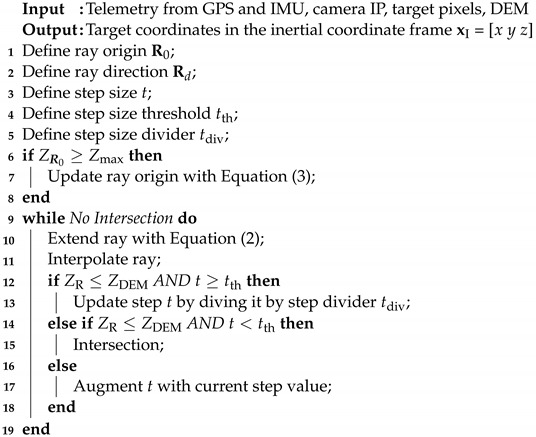


### 3.4. Uncertainty Characterization with the Unscented Transform

Three sources of uncertainty were taken into account: the vehicle GPS, IMU and gimbal. The GPS contributed with three degrees of uncertainty related to the position of the vehicle in the vehicle frame, $\sigma_{x}$, $\sigma_{y}$ and $\sigma_{z}$. The vehicle IMU contributed with three degrees of uncertainty related to the orientation angles of the vehicle with respect to the vehicle frame, roll $\sigma_{\phi }$, pitch $\sigma_{\theta }$, and yaw $\sigma_{\psi }$. Finally, the gimbal contributed with two degrees of uncertainty related to the elevation and azimuth angles that establish the orientation of the gimbal with respect to the body frame, $\sigma_{\alpha_{el}}$ and $\sigma_{\alpha_{az}}$. This makes a total of n=8 degrees of uncertainty. The standard deviations presented in Table 1 follow the guidelines in [21] for typical values of the telemetry errors. The different coordinates are assumed independent, and the covariance matrix is defined as

$$\Sigma =\mathrm{diag}(\sigma_{x}^{2},\sigma_{y}^{2},\sigma_{z}^{2},\sigma_{\phi }^{2},\sigma_{\theta }^{2},\sigma_{\psi }^{2},\sigma_{\alpha_{el}}^{2},\sigma_{\alpha_{az}}^{2})$$

The UT parameters were set according to Table 2. Since we approximate the distribution as a Gaussian, β = 2 is the optimal choice to minimize higher order information from the Taylor Series expansion. As for α and κ, these values were chosen so as to have the sigma points equal to the standard deviations of the equipment.

The target position and uncertainty are calculated by propagating the sigma points with the complete iterative ray-tracing using the Algorithm 2:
**Algorithm 2:** Unscented Transform with IRT.
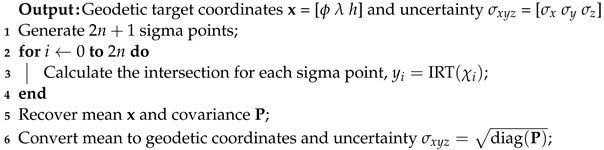


### 3.5. Bearings-Range Measurement Model

Considering measurement errors in the GPS and IMU, this section details the proposed vision-based target localization method using using a filtering strategy on bearings and range measurements. Previous works using filtering approaches in the georeferencing problem [20,21] use a bearings-only approach. In this paper, we propose a new filter measurement model that takes advantage of the available range information between the vehicle and the target computed by Iterative Ray-Tracing under the availability of a DEM of the observed area. Experiments will then compare the new Bearings-Range filter with the more common Bearings-Only filter, to assess the benefit of the additional depth information computed by IRT. Taking into account the possible linearization errors induced by the EKF, a performance comparison is also done with a CKF.

**Figure 2:**
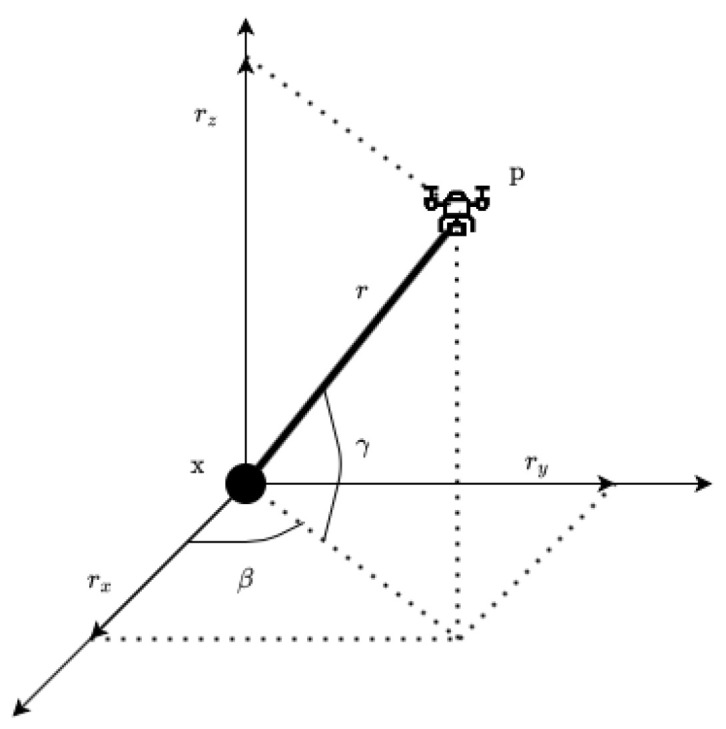
Azimuth (β), elevation (ϕ) and range (*r*) between vehicle (p) and target (x).

The bearings localization problem is based on the extraction of the azimuth β and elevation γ angles from the pointing vector, as shown in Figure 2. Since the proposed georeferencing algorithm calculates the 3D coordinates of the target with the support of a DEM, it enables the estimation of the distance *r* between the vehicle and the target.

The measurement model $h(x_{k})$ we propose is given by

$$h\left(x_{k}\right)=\left[\begin{array}{c} \beta \\ \gamma \\ r \end{array}\right]=\left[\begin{array}{c} \tan^{-1}\left(\frac{r_{y}}{r_{x}}\right)\\ \tan^{-1}\left(\frac{r_{z}}{\sqrt{r_{x}^{2}+r_{y}^{2}}}\right)\\ \sqrt{r_{x}^{2}+r_{y}^{2}+r_{z}^{2}} \end{array}\right]$$

where $r_{k}={\left[r_{x}\;r_{y}\;r_{z}\right]}_{k}^{T}={\left[p_{x}-x_{x},\;p_{y}-x_{y},\;p_{z}-x_{z}\right]}_{k}^{T}$ is the relative vector between the vehicle and the target for the kth measurement, $p_{k}={\left[p_{x}\;p_{y}\;p_{z}\right]}_{k}^{T}$ is the position of the vehicle and $x_{k}={\left[x_{x}\;x_{y}\;x_{z}\right]}_{k}^{T}$ is the position of the target and the state to be estimated.

Assuming that the target is stationary (in our problem, the displacement of a fire front is small in the time frame of the filtering process), the target dynamics model is given by

$$f\left(x_{k}\right)=\Phi_{k+1|k}x_{k}=\left[\begin{array}{ccc} 1 & 0 & 0\\ 0 & 1 & 0\\ 0 & 0 & 1 \end{array}\right]x_{k}$$

and the covariance of the system noise $w_{k}$ is given by

$$Q_{k}=\left[\begin{array}{ccc} 0 & 0 & 0\\ 0 & 0 & 0\\ 0 & 0 & 0 \end{array}\right]$$

For the EKF, the Jacobian of the measurement function with respect to the state is

$$H_{k}={\left[\begin{array}{ccc} \frac{r_{y}}{r_{x}^{2}+r_{y}^{2}} & -\frac{r_{x}}{r_{x}^{2}+r_{y}^{2}} & 0\\ \frac{r_{x}r_{z}}{{\left||r|\right|}^{2}\sqrt{r_{x}^{2}+r_{y}^{2}}} & \frac{r_{y}r_{z}}{{\left||r|\right|}^{2}\sqrt{r_{x}^{2}+r_{y}^{2}}} & -\frac{\sqrt{r_{x}^{2}+r_{y}^{2}}}{{\left||r|\right|}^{2}}\\ -\frac{r_{x}}{\left||r|\right|} & -\frac{r_{y}}{\left||r|\right|} & -\frac{r_{z}}{\left||r|\right|} \end{array}\right]}_{k}$$

The sensor noise mentioned in Section 3.4 is used to model the noise covariance matrix, tuned to the following values

$$R_{k}=\left[\begin{array}{ccc} \sigma_{\alpha_{az}}^{2} & 0 & 0\\ 0 & \sigma_{\alpha_{el}}^{2} & 0\\ 0 & 0 & \sigma_{r}^{2} \end{array}\right]=\left[\begin{array}{ccc} 1^{2} & 0 & 0\\ 0 & 1^{2} & 0\\ 0 & 0 & 10^{2} \end{array}\right]$$

where $\sigma_{\alpha_{az}}{[}^{\circ }]$ and $\sigma_{\alpha_{el}}{[}^{\circ }]$ are the gimbal’s azimuth and elevation uncertainties and $\sigma_{r}\left[m\right]$ is the range uncertainty. The IRT and UT results of the first observation initialize the filters’ state and covariance, ${\hat{x}}_{0}$ and ${\hat{P}}_{0}$.

### 3.6. Metrics

The metrics presented here will be used throughout the following sections. The position error

$$e_{p}={\left[x_{x}\;x_{y}\;x_{z}\right]}^{T}-{\left[{\hat{x}}_{x}\;{\hat{x}}_{y}\;{\hat{x}}_{z}\right]}^{T}$$

is used to determine the distance between the target position, *x*, and its estimates, $\hat{x}$. To characterize the accuracy of the algorithm, the average position error (Equation (4)) and RMSE (Equation (5)) are defined by, respectively

(4) $$\mu_{e_{p}}=\frac{\sum_{i=1}^{N}\sqrt{e_{p_{x_{i}}}^{2}+e_{p_{y_{i}}}^{2}+e_{p_{z_{i}}}^{2}}}{N},$$

(5) $$RMSE=\sqrt{\frac{\sum_{i=1}^{N}e_{p_{x_{i}}}^{2}+e_{p_{y_{i}}}^{2}+e_{p_{z_{i}}}^{2}}{N}}.$$

Although the former provides the average distance between the estimates and the target, the latter characterizes their distribution.

The target estimate uncertainty $\sigma_{xyz}$ is given by

$$\sigma_{xyz}={\left[\sigma_{xx},\;\sigma_{yy},\;\sigma_{zz}\right]}^{T}$$

based in the square root of the diagonal values of the target estimate covariance matrix P

$$P=\left[\begin{array}{ccc} \sigma_{xx}^{2} & \sigma_{xy}^{2} & \sigma_{xz}^{2}\\ \sigma_{yx}^{2} & \sigma_{yy}^{2} & \sigma_{yx}^{2}\\ \sigma_{zx}^{2} & \sigma_{zy}^{2} & \sigma_{zz}^{2} \end{array}\right].$$

Finally, the average position uncertainty is defined by

$$\mu_{\sigma_{xyz}}=\frac{\sum_{i=1}^{N}\sqrt{\sigma_{xx_{i}}^{2}+\sigma_{yy_{i}}^{2}+\sigma_{zz_{i}}^{2}}}{N}.$$

## 4. Simulation Experiments

For the evaluation of the developed methods with ground truth, we performed graphical simulations with two types of terrains—rough and flat—as in previous works on this topic [21]. In both cases, a linear trajectory was followed, with the aerial vehicle flying at a speed of 250 km/h. Ground truth telemetry was generated and perturbed with the following model:

(6) $$\hat{x_{i}}\left(t\right)=x_{i}\left(t\right)+w\left(t\right),\;w∼N\left(0,\sigma_{x_{i}}\right),$$

where ${\hat{x}}_{i}$ is the noisy variable, $x_{i}$ is the ground truth variable, w is the zero-mean noise with $\sigma_{x_{i}}$ standard deviation. The assumed values for $\sigma_{x_{i}}$ are the same as the ones used for the UT, presented in Table 1. For each simulation, 100 runs were performed with independent noise sequences, and the EKF and CKF performances using the Bearings-Range (BR-EKF, BR-CKF) and Bearings-Only (BO-EKF, BO-CKF) measurement models were compared. The results presented are an average of all the runs.

### 4.1. Rough Terrain Simulation

**Figure 3:**
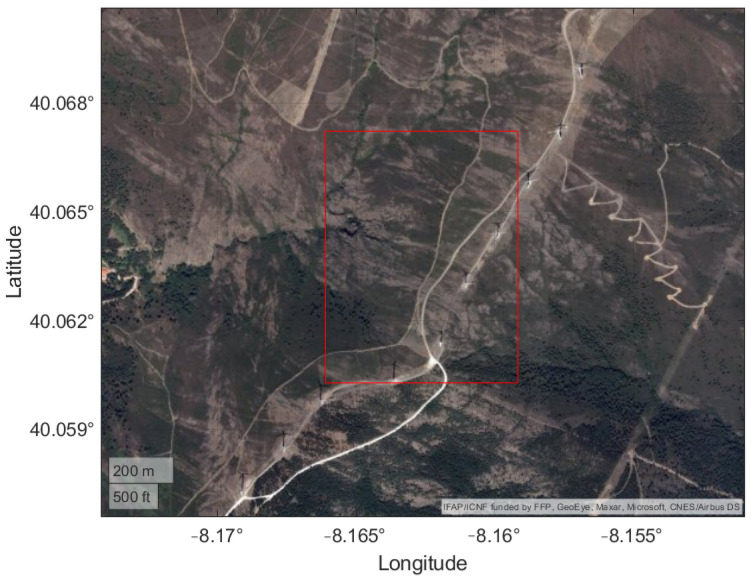
Satellite view of the testing area near *Coentral, Leiria*.

**Figure 4:**
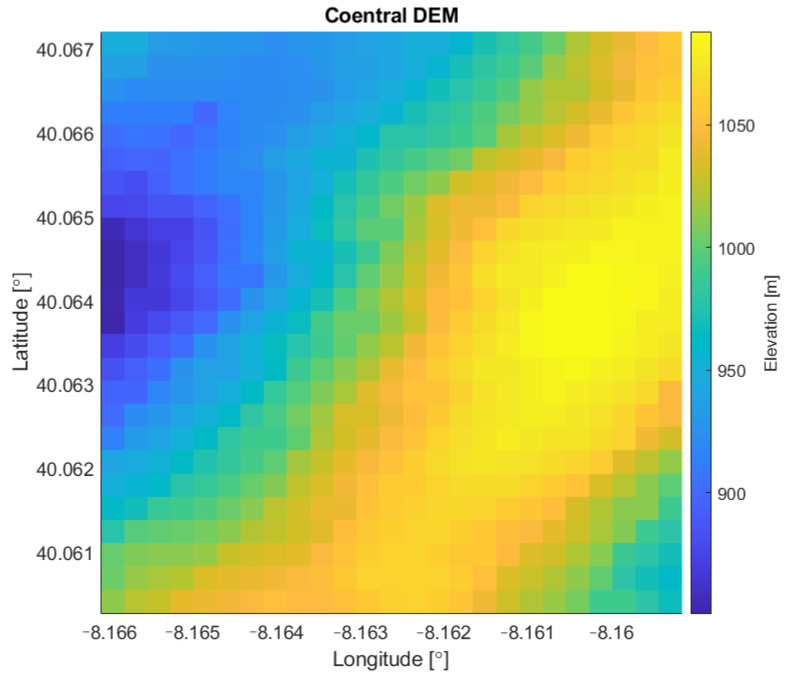
DEM of testing area near *Coentral, Leiria*.

The chosen rough terrain is located near Coentral, Leiria, Portugal. The main characteristics and geographical coordinates of the terrain are summarized in Table 3. Figure 3 shows the chosen rough terrain area and the respective DEM is presented in Figure 4.

Simulations were performed in the Gazebo Simulator (http://gazebosim.org, accessed on 15 September 2021) that allowed us to acquire telemetry and imagery data. Gazebo is an open-source robot simulator useful for simulating robots with a diversity of sensors and actuators. In the context of this work, it was used to acquire the position and orientation of a vehicle and imagery of a target using a camera attached to said vehicle. A linear trajectory parallel to the terrain elevation was simulated, with constant yaw equal to 48${}^{\circ }$, roll and pitch equal to 0${}^{\circ }$, and a variable azimuth and elevation angles that kept the target in sight of the camera. This way, it was possible to manually identify the pixel corresponding to the target on each image. Because there is no fire model available in the Gazebo library, a checkerboard was used to represent the target.

**Figure 5:**
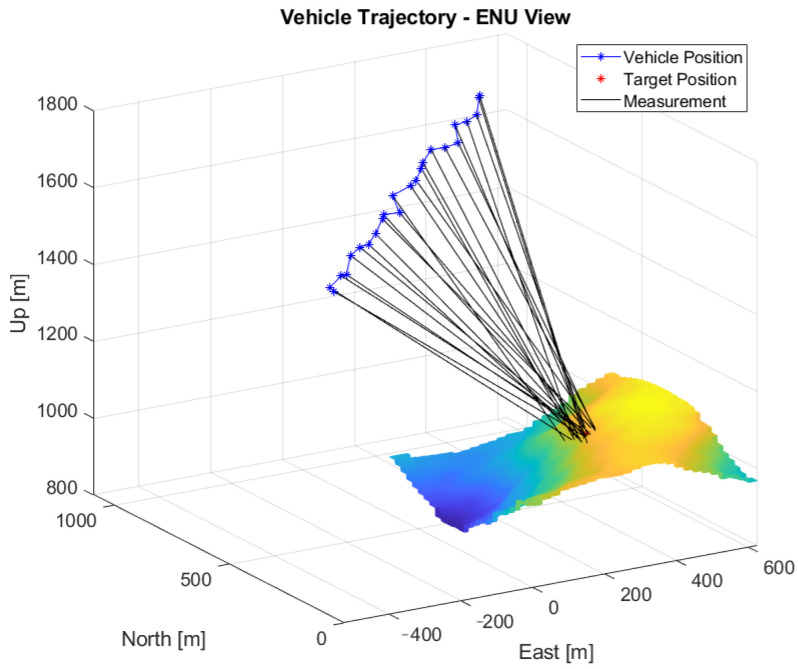
Measurements with noise-induced position and attitude on the rough terrain simulation.

**Figure 6:**
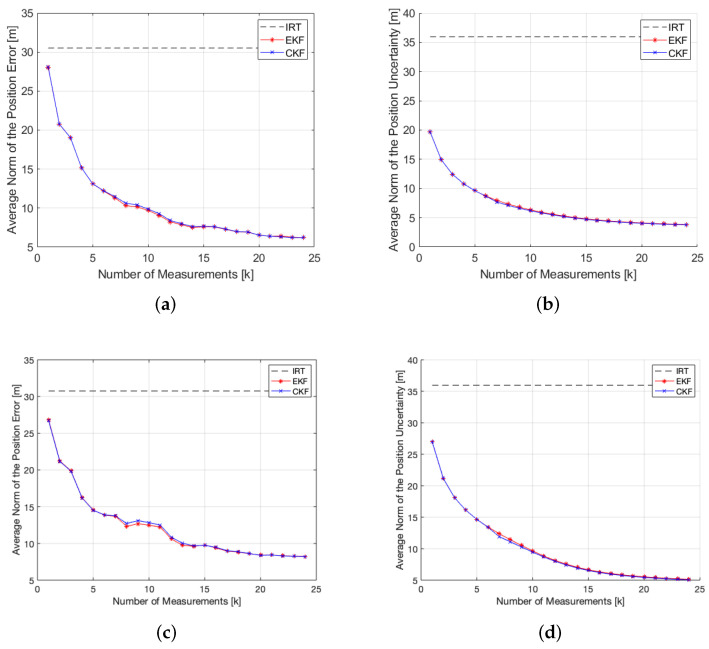
EKF and CKF filtering results for the rough terrain simulation. (**a**) BR-EKF and BR-CKF average norm of the position error; (**b**) BR-EKF and BR-CKF average norm of the position uncertainty; (**c**) BO-EKF and BO-CKF average norm of the position error; (**d**) BO-EKF and BO-CKF average norm of the position uncertainty.

A total of 25 measurements were collected at a constant height of 1650 m. Figure 5 shows the resulting measurements following the model presented in Equation (6). The average distance to the target was 880 m. Figure 6 shows the evolution of the position error and uncertainty with the number of measurements for the Bearings-Range and Bearings-Only models with the EKF and CKF filters. Table 4 shows the numerical results of the average position errors, RMSE and average position uncertainty at the end of the filtering process.

### 4.2. Flat Terrain Simulation

**Figure 7:**
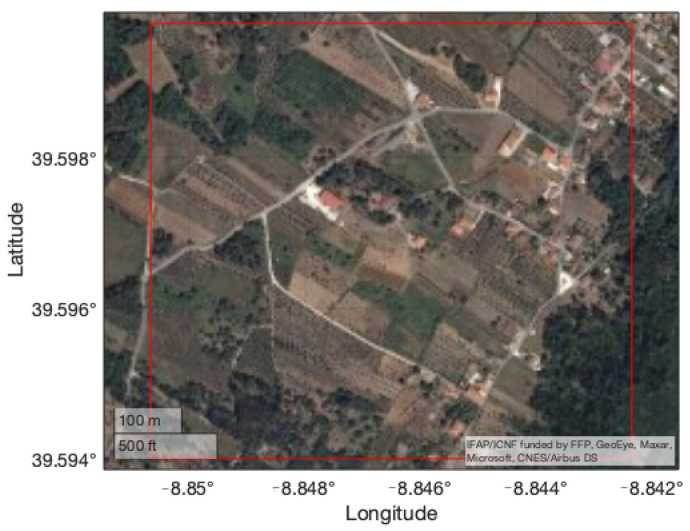
Satellite view of the testing area near *Porto de Mós, Leiria*.

**Figure 8:**
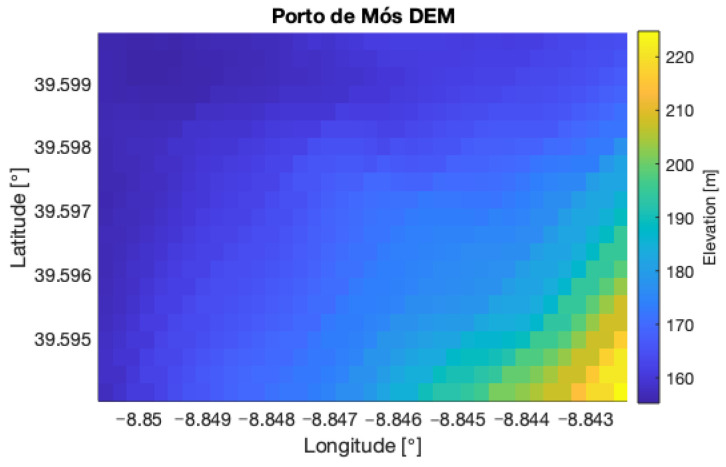
Testing area near *Porto de Mós, Leiria*.

Regarding the flat terrain dataset, a small area near Porto de Mós, Leiria, Portugal, represented in Figure 7, was chosen. The main characteristics and geographical coordinates of the area are summarized in Table 5 and its DEM is presented in Figure 8. The same area was later used to perform a real experiment, to be presented in the following section.

For this terrain type, MATLAB was used to generate ground truth telemetry data. As in the previous data set, a linear trajectory was simulated. This time, the vehicle moved East, therefore with the yaw equal to 90${}^{\circ }$, while the roll and pitch were set to 0${}^{\circ }$. It was assumed that the target was always centered in the images, so no camera model was needed and the azimuth and elevation angles were calculated based on the trigonometric relation between the aerial vehicle and the target.

**Figure 9:**
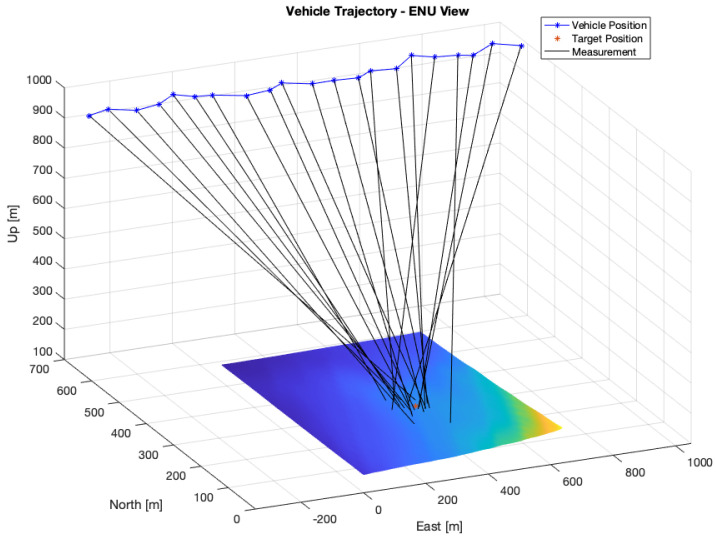
Measurements with noise-induced position and attitude on the flat terrain simulation.

A total of 21 measurements were collected at a constant height of 950 m. Figure 9 shows the resulting measurements with noisy data. The average distance to the target was 985 m.

### 4.3. Discussion of Simulation Results

**Figure 10:**
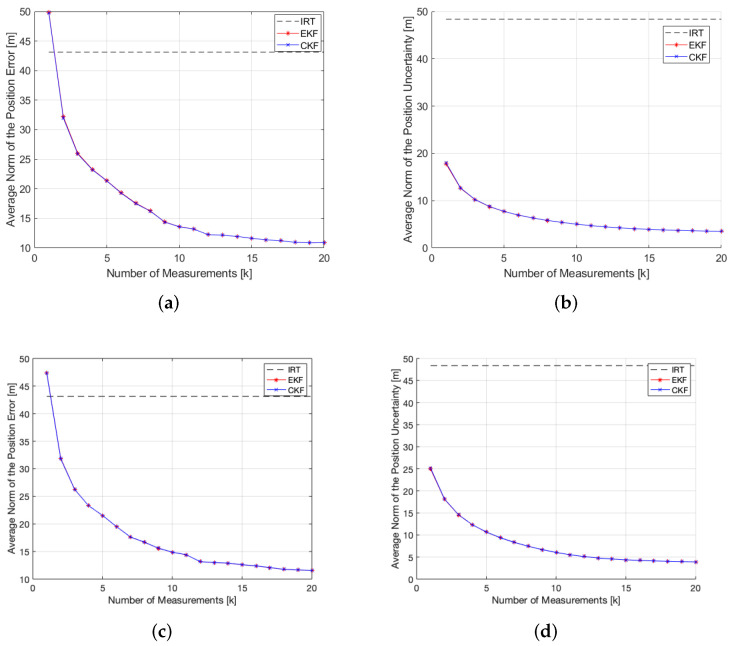
EKF and CKF filtering results for the flat terrain simulation. (**a**) BR-EKF and BR-CKF average norm of the position error; (**b**) BR-EKF and BR-CKF average norm of the position uncertainty; (**c**) BO-EKF and BO-CKF average norm of the position error; (**d**) BO-EKF and BO-CKF average norm of the position uncertainty.

These simulations demonstrate the advantage of including the range information in the filtering algorithm. Both in the rough and flat simulations, the Bearings-Range measurement model achieves lower position errors, position uncertainties and RMSE’s for the same number of measurements, therefore is more accurate than the Bearings-Only measurement model. Furthermore, it has a faster convergence, evident for k=10 in Figure 6 and Figure 10. The results presented in Table 4 and Table 6 show the clear improvement on the accuracy of the estimated target position when applying the Bearings-Range filtering algorithm versus the standalone IRT. The RMSE is reduced by 61.86% and 54.12% for the rough and flat terrains, respectively, both for the BR-EKF and BR-CKF. For both achieve the same final result with an identical progression in the position error and uncertainty, there is no clear advantage in using the BR-CKF over the BR-EKF. In addition, the EKF shows a slightly faster processing time (Table 7), making it more appropriate for real-time applications.

## 5. Results with Real Footage

To test the effectiveness of the proposed algorithm with real data, two experiments were designed.

Section 5.1 details the procedure where a mobile phone was used to acquire telemetry and imagery of a target along a pedestrian path, on a hilly terrain near *Porto de Mós*, *Leiria*, *Portugal*. This experiment allows the verification of the effect of low-viewing angles to the target. Low angle perspectives can happen in the observation of wildfires from drones. Drones cannot fly directly above the wildfire due to smoke and heat convection airflow, so they must take observations from lateral perspectives.

Two simulations, along the same XY coordinates but at different heights were also performed. This allowed us to assess the dependency of the georeferencing method with the view angle.

In Section 5.2 the algorithm is applied to a footage recorded by a Portuguese Air Force UAV near *Chaves*, *Vila Real*, *Portugal*, in a real firefighting scenario.

Finally, in Section 5.3, we perform and experiment using GCP. The GCP are used to obtain precise estimates of the camera pose, correcting telemetry errors. This experiment thus defines an upper bound to the achievable performance of the standalone IRT georeferencing method. This method, because it uses GCP, cannot be considered a direct georeferencing method, and cannot be applied for real-time georeferencing in forest scenarios due to the difficulty in acquiring reliable landmarks automatically in such scenarios. However, it allows us to figure out the achievable performance of standalone IRT as if noise in IMU and GPS could be neglected. This experiment is made with real footage recorded near *Pombal*, *Leiria*, *Portugal*, in a firefighting scenario in a location where several GCP could be identified in a nearby village. An optimization problem is formulated to minimize the reprojection error of the GCP to calculate the camera’s intrinsic parameters and rotations, which are then used in the georeferencing algorithm.

### 5.1. Mobile Phone Procedure

**Figure 11:**
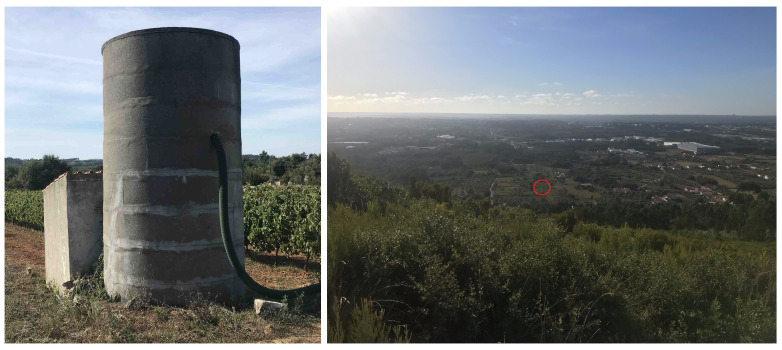
Structure used as target (**left**) and one of the photos used in the georeferencing procedure, with the target marked with a red circle (**right**).

A mobile phone was used to record GPS, IMU and image data along a pedestrian path. The natural elevation of *Serra dos Candeeiros*, near *Porto de Mós*, *Leiria*, *Portugal* was used to capture images of a target (Figure 11) located at a lower height.

A total of 14 images were acquired at an average camera-to-target distance of 605 m. For this experiment, considering that the IMU of the mobile phone had a lower quality when compared to the ones used onboard an aerial vehicle, the measurement noise covariance matrix $R_{k}$ was tuned to

$$R_{k}=\left[\begin{array}{ccc} 5^{2} & 0 & 0\\ 0 & 5^{2} & 0\\ 0 & 0 & 10^{2} \end{array}\right].$$

The camera was calibrated using a dataset of 20 images. As a result, the following intrinsic and distortion parameters were obtained:

$$K_{\mathrm{int}}=\left[\begin{array}{ccc} 3363.507 & 0 & 1967.377\\ 0 & 3369.501 & 1419.890\\ 0 & 0 & 1 \end{array}\right]$$

$$k={\left[\begin{array}{ccc} 0.2265 & -1.0227 & 1.7296 \end{array}\right]}^{T}$$

$$p={\left[\begin{array}{cc} -0.0098 & -0.0065 \end{array}\right]}^{T}$$

A mean reprojection error of 0.68 pixels was achieved with a 3-parameter radial distortion model.

**Figure 12:**
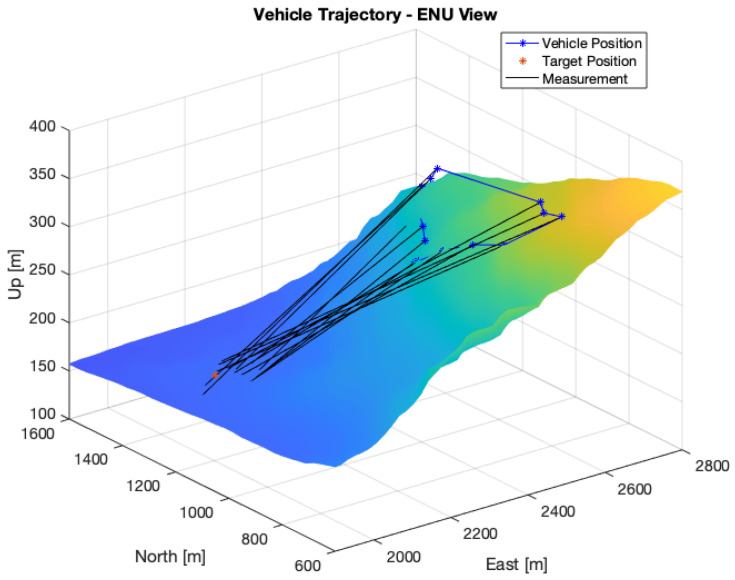
Trajectory and IRT results in local ENU coordinate system.

In Figure 12 the trajectory is plotted in a local ENU coordinate system, with the location of the images, the target and the estimated locations of the target. The average distance between the image positions and the target was 640.83 m, with a maximum of 770.92 m and 493.90 m. The results are presented in Table 8.

**Figure 13:**
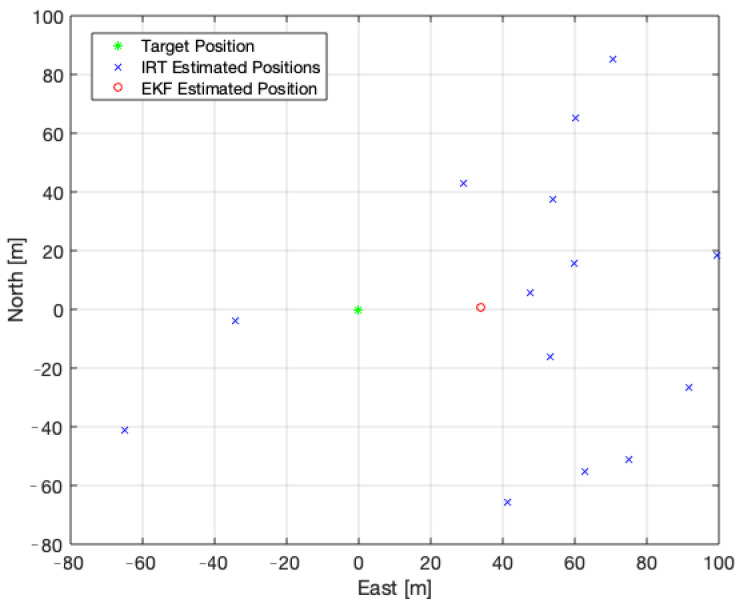
Real position of the target (green), IRT measurements (blue) and estimated position with the EKF (red).

Figure 13 provides a clear view of the measurements obtained with the IRT and the improvement achieved by applying the BR-EKF. A bias along the positive East direction is visible, since 12 of the 14 IRT measurements are in that area.

**Figure 14:**
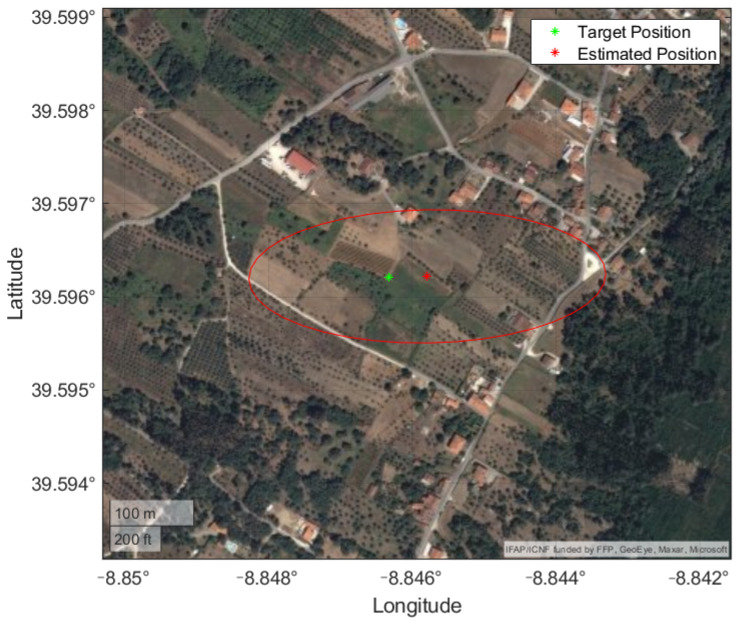
Mean position and uncertainty from experimental procedure with real data displayed on a satellite view of the terrain.

This can be due to the non-ideal experimental setup, i.e., a line-of-sight more parallel to the ground when compared to a more vertical one from an aerial vehicle. The mean position and uncertainty are plotted in Figure 14 and evidence this ill-conditioned setup, with a stretched uncertainty region along the line-of-sight direction. This means that a small error on the vertical image direction, be it the selected pixel or the roll angle, is amplified by this configuration. In addition, the images were taken at approximate positions, limiting the new information added to the BR-EKF.

#### 5.1.1. Simulations from Ground Perspective

**Figure 15:**
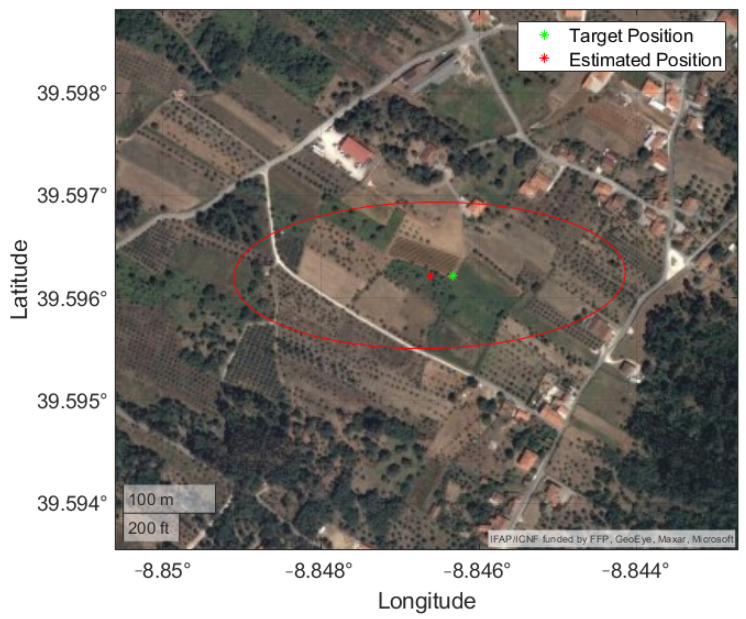
Mean position and uncertainty from ground perspective simulation displayed on a satellite view of the terrain.

The same experiment was simulated to investigate if the ill-conditioning observed with the real data was due to the non-ideal perspective or pixel measurement errors (the target pixel coordinates were selected manually). A simulation was performed with the same trajectory but with the camera orientations computed to center the target with the image origin, to cancel pixel measurement errors. Noise to GPS and IMU was added according to Table 1. The mean position and uncertainty are plotted in Figure 15. The results are presented in Table 9 and Figure 15. The uncertainty ellipsoid in Figure 15 still shows an East-West elongation, reflecting the ill-conditioning of the problem even without pixel measurement errors. In fact, the simulated results are worse than with real data, with an increase of almost 100% in the RMSE and 72 m in the position uncertainty when compared to Table 8, suggesting that the telemetry noise added in simulation may be larger than in the real data. Furthermore, the mean estimated target position presented Figure 15 is shifted towards West, instead of what happened with real data (East), suggesting that the source of the bias is of random nature.

#### 5.1.2. Simulation from Aerial Perspective

**Figure 16:**
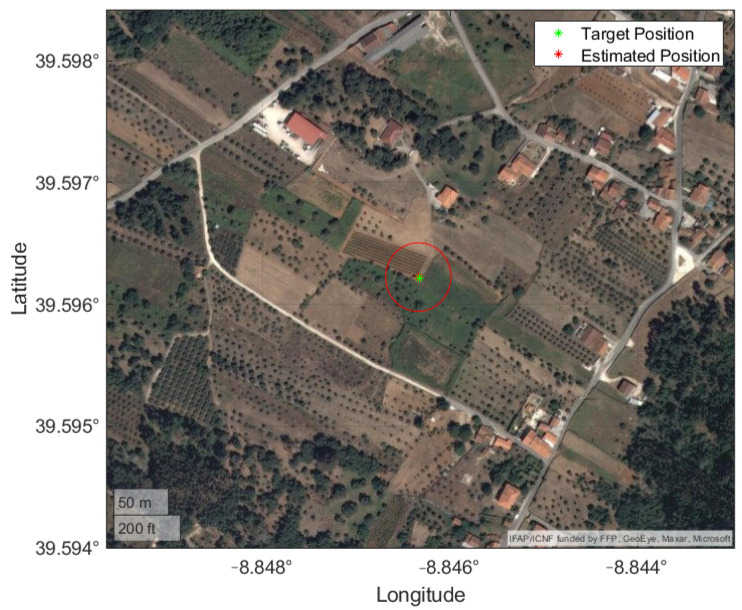
Mean position and uncertainty from aerial perspective simulation displayed on a satellite view of the terrain.

Finally, the same trajectory was simulated with an aerial perspective at a height of 950 m. The results presented in Table 10 show that higher perspectives are beneficial to the quality of the results, as expected. The RMSE decreased by 36 m and the position uncertainty was reduced by 113 m, as a result of a more perpendicular line-of-sight demonstrated in Figure 16. In addition, the biases identified in the experimental procedure and ground simulation are no longer present since the mean estimated target position is practically identical to the real target position. In conclusion, the use of more tilted perspective is clearly advantageous when applying the proposed algorithm, providing a more accurate estimate of the target’s position, which is further improved with the Bearings-Range EKF.

### 5.2. Portuguese Air Force UAV Footage

**Figure 17:**
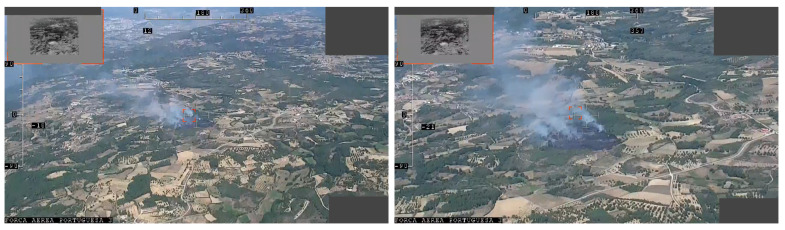
Sample frames of the Chaves video.

A UAV operated by the Portuguese Air Force recorded a fire burning video near *Chaves*, *Vila Real*, *Portugal*, at 41.631724 N −7.465919 E. A section of this video was gently provided by the Portuguese Air Force for this research. A couple of video frames from this video are shown in Figure 17. The video feed metadata contains the UAV’s position in the WGS84 reference system and the gimbal azimuth and elevation angles. A total of 7 frames were extracted and used to test the algorithm.

The used camera is a *Sony EV-7500* with a lens of 30× optical zoom, f=4.3 mm to f=129 mm (F 1.6 to F 4.7, HFOV 63.7). Despite the intrinsic parameters being known, the camera zoom level at the recording time is not specified. However, since the target was locked in the center of the image, it was possible to perform the georeferencing algorithm without (he knowledge of the focal distance we assumed the principal point did not change with the zoom level, so the optical ray coincides with the Z coordinate of the camera reference frame, independently of the focal distance).

**Figure 18:**
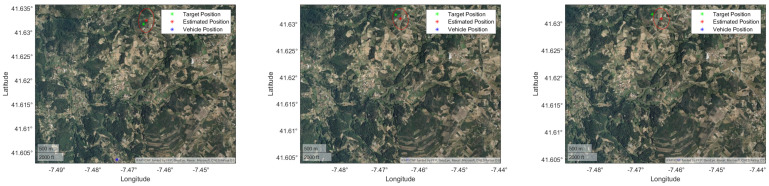
Portuguese Air Force footage georeferencing results displayed on a satellite view of the terrain. The red marks and ellipses show, respectively, the IRT measurement and the estimated uncertainty computed by the UT in three sample frames of the video.

The UAV flew at approximately 1920 m, and the average distance to the target was 3183 m, meaning that the horizontal component was 2539 m. The results are presented in Table 11 and Figure 18. We can observe that the filtering strategy, in this case, did not improve on the results of the unfiltered IRT. As in the *Porto de Mós* experimental procedure, the viewing perspective was low. This fact made the observations very similar, thus, not much information was added on each EKF iteration. Furthermore, only 7 measurements were available, where one was used to initialize the filter state and covariance and only six were left to iterate.

### 5.3. UAVision UAV Footage

**Figure 19:**
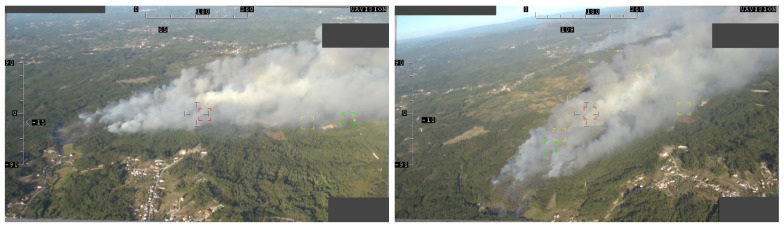
Sample frames of the Pombal video.

In this experiment we used a recorded video of a forest fire near *Pombal*, *Leiria*, at 39.832856 N −8.519885 E gently provided by UAVision (uavision.com accessed on 15 September 2021). The camera and video characteristics are the same as in the previous experiment A couple of video frames from this video are shown in Figure 19.

The video covered a forest area close to small villages where GCP could be easily identified. Three video frames with visible landmarks and known coordinates were selected. This allowed the formulation of a minimization problem to estimate the intrinsic parameters and to refine the rotation matrix of the system when those images were captured.

As a cost function, the reprojection error was used

(7) $$J\left(R_{i},t_{i},K_{int}\right)=\sum_{i=1}^{3}\sum_{k=1}^{N_{k}}{\left(u_{ik}-{\hat{u}}_{ik}\right)}^{2}+{\left(v_{ik}-{\hat{v}}_{ik}\right)}^{2},$$

where $R_{i}$ and $t_{i}$ are the rotation matrix and translation vector that establish the transformation from world to camera coordinates for frame *i* (initialized from the vehicle telemetry), $K_{\mathrm{int}}$ is the IP matrix (initialized from the camera defaults at the widest zoom level – f=4.3mm), $\left(u_{ik},v_{ik}\right)$ is the measured pixel *k* in frame *i* and $\left({\hat{u}}_{ik},{\hat{v}}_{ik}\right)$ is the predicted pixel using the current estimates of $R_{i}$, $t_{i}$ and $K_{\mathrm{int}}$. It was assumed that the skew *s* was zero, $f_{x}=f_{y}$ and $c_{x}=640$ and $c_{y}=360$. The optimization problem to solve is:

(8) $$\begin{array}{cc} \arg\;\min J(R_{i},t_{i},K_{\mathrm{int}})\\ s.t.\; & \left|\right|r_{i1}\left|\right|=1,\;||r_{i2}\left|\right|=1,\;||r_{i3}\left|\right|=1\\ \; & r_{i1}r_{i2}^{T}=0,\;r_{i2}r_{i3}^{T}=0,\;r_{i1}r_{i3}^{T}=0\\ \; & f_{x}=f_{y} \end{array},$$

where $r_{ik}$ is the row *k* of rotation matrix *i*. The six initial constraints are related to the orthogonality condition of the rotation matrices.

The estimated intrinsic parameters from the minimization problem were

(9) $$K_{\mathrm{int}}=\left[\begin{array}{ccc} 1063.17 & 0 & 640\\ 0 & 1063.17 & 360\\ 0 & 0 & 1 \end{array}\right].$$

These parameters and the refined rotation matrices were used to georeference the position of several landmarks. Table 12 presents the number of landmarks selected in each frame and the average norm of the position error, average norm of position uncertainty and RMSE.

**Figure 20:**
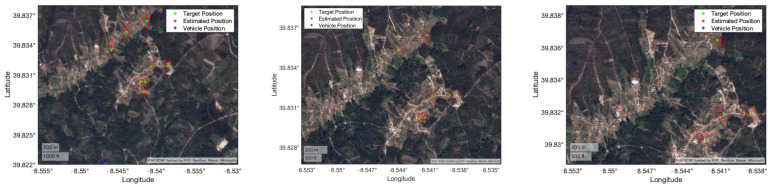
UAVision georeferencing results displayed on a satellite view of the terrain. The red marks and ellipses show, respectively, the IRT measurement and the estimated uncertainty computed by the UT in three sample frames of the video.

Frames 1 and 2 present similar results in terms of position error and RMSE. Position uncertainty is greater on the first frame because it is georeferencing targets that are further from the vehicle than in the second frame. The third and final frame presents the worst results in terms of position error and RMSE. This is due to being the image with less landmarks, and consequently is less refined by the minimization problem when compared to the other frames. Overall, accurate results were obtained for targets that distanced more than one kilometer from the UAV, as shown in Figure 20. In a real scenario, where the position error and RMSE will not be available, the uncertainty is taken into account as metric of the georeferencing algorithm, with a lower value representing a more trustworthy estimated position.

### 5.4. Discussion of Experimental Results

The IRT benefits from using an aerial perspective, as demonstrated in the first experiment. With this approach, the line-of-sight is more perpendicular to the terrain, therefore errors in the system attitude are less amplified. The results presented in Table 9 and Table 10 show a decrease of the mean position error, RMSE and mean position uncertainty of 64.74%, 66.06% and 81.03%, respectively. With more accurate IRT measurements, the BR-EKF also achieves a more accurate target position with a RMSE of 21.946 m when compared to the 94.686 m obtained with the ground simulation.

In contrast with the first experiment, the results of the second experiment were taken at a line-of-sight more parallel to the ground and a larger distance to the target that significantly increased the magnitude of the position uncertainty to 292.6 m (Table 11). Furthermore, the reduced number of measurements and the lack of spatial variability in the UAV’s position prevented the filtering algorithm from improving the target coordinates. To surpass these limitations, more measurements are required. Additionally, these measurements should be captured from more diverse positions to provide more information to the BR-EKF.

Lastly, the final experiment shows the need for accurate telemetry and target identification. Applying the minimization problem of Equation (8) to each frame allowed us to determine the unknown camera’s IP and refine the UAV telemetry. With these, the standalone IRT was able to achieve accurate target positions, with an average RMSE of 16.439 m on the three frames (Table 12) for target distances greater than 1000 m. We note, however, that this method required the identification of GCP which is difficult to automate, particularly in forest scenarios where landmarks are not frequent.

## 6. Conclusions

In this paper, we propose a method to georeference targets in forest environments from videos acquired at aerial perspectives. The fact that forest environments are not rich in landmarks, prevents the use of ground control points to perform the geolocalization. Thus, we have developed a direct georeferencing method combining best practices on the state of the art, and evaluate it both in simulations and with real footage, in a diversity of situations, including real firefighting scenarios. Our best method can be summarized as an Iterative Ray-Tracing, Bearings-Range Extended Kalman Filter, where the measurements are obtained from GPS, IMU and Camera data, supported by a Digital Elevation Map of the observed area. The IRT algorithm proposed in this work presents a robust solution for forest fire georeferencing. It only requires the camera’s IPs, the onboard sensor data (GPS/IMU/gimbal) and a DEM, while current state-of-the-art approaches rely on feature identification and matching. Equipped with the UT, the georeferencing algorithm provides an estimate of the target position and characterizes its uncertainty. That is a relevant achievement since it provides crucial information regarding the confidence of the estimated target position to initialize the filtering process, to feed eventual stochastic simulations of fire propagation, and, ultimately, to inform decision support systems to the firefighting personnel.

The results obtained in a controlled simulation environment demonstrate the potential of the developed algorithm. At distances of almost 1000 m, two simulations of the raw IRT method were run on rough and flat terrains, achieving RMSEs of 30.7 and 43.4 m with 25 and 21 measurements, respectively. After applying the BR-EKF, these errors reduced to 11.0 and 19.9 m, a decrease of 58.5% and 54.7%. This relation was also verified in the experiments with real data. In the mobile phone experimental procedure, an initial RMSE of 77.5 m was reduced to 41.5 m with just 14 measurements. Finally, georeferencing with accurate intrinsic and extrinsic parameters, as was the case in the UAVision experimental procedure, resulted in accurate estimated positions. An average RMSE of 16.33 m was achieved with a total of 18 landmarks that distanced more than 1000 m from the UAV. Although this method requires ground control points, not always available in forest environments, it shows the potential of the proposed methods, provided good telemetry data.

### Future Work

The experiment with the Air Force footage exposed the main limitations of the proposed method. Very distant targets and tilted perspectives make the IRT measurements noisy. On top of that, if video sequences are short and of low variability in perspective, the filter has trouble in improving the raw IRT measurements. Therefore, it is important to assure rich enough camera trajectories. A possible next step would be to design flight patterns that minimize the error of the IRT algorithm, but also maximize the information extracted from the Bearings-Range measurement model. In addition, considering the last experimental procedure, an algorithm could be developed that searches recognizable landmarks in the imagery acquired by the aerial vehicle, whenever they are available, to improve the camera calibration.

## Figures and Tables

**Table 1:** GPS, IMU and Gimbal standard deviations.

Device	Standard Deviation $\sigma_{i}$	Value
GPS	$\sigma_{x}$ $\sigma_{y}$ $\sigma_{z}$	10 m 10 m 10 m
IMU	$\sigma_{\phi }$ $\sigma_{\theta }$ $\sigma_{\psi }$	1${}^{\circ }$ 1${}^{\circ }$ 3${}^{\circ }$
Gimbal	$\sigma_{\alpha_{el}}$ $\sigma_{\alpha_{az}}$	1${}^{\circ }$ 1${}^{\circ }$

**Table 2:** UT Parameters.

Parameter	Value
α	$\frac{1}{\sqrt{8}}$
κ	0
β	2

**Table 3:** *Coentral* DEM characteristics.

Characteristics	
Maximum Height [m]	1088.00
Minimum Height [m]	850.57
WGS84/EPSG:4326 Coordinates [${}^{\circ }$]	N[40.060, 40.067] E[−8.166,−8.159]

**Table 4:** IRT, IRT+BR-EKF, IRT+BO-EKF, IRT+BR-CKF and IRT+BO-CKF results for the rough terrain scenario.

Method	$\mu_{e_{p}}$ [m]	RMSE [m]	$\mu_{\sigma_{\mathrm{xyz}}}$ [m]
IRT	30.527	30.743	35.965
IRT+BR-EKF	6.221	11.726	3.837
IRT+BO-EKF	8.225	13.071	5.137
IRT+BR-CKF	6.222	11.785	3.782
IRT+BO-CKF	8.193	13.136	5.054

**Table 5:** *Porto de Mós* DEM characteristics.

Characteristics	
Maximum Height [m]	224.82
Minimum Height [m]	155.25
WGS84/EPSG:4326 Coordinates [${}^{\circ }$]	N[39.5940, 39.5998] E[−8.8507,−8.8424]

**Table 6:** IRT, IRT+BR-EKF, IRT+BO-EKF, IRT+BR-CKF and IRT+BO-CKF results for the flat terrain scenario.

Method	$\mu_{e_{p}}$ [m]	RMSE [m]	$\mu_{\sigma_{\mathrm{xyz}}}$ [m]
IRT	43.121	43.405	48.373
IRT+BR-EKF	11.020	19.910	3.898
IRT+BO-EKF	11.565	21.578	3.909
IRT+BR-CKF	11.000	19.842	3.899
IRT+BO-CKF	11.575	21.573	3.911

**Table 7:** EKF and CKF processing time comparison.

Kalman Filter	Average Processing Time [ms]
Extended	0.795
Cubature	0.83

**Table 8:** Standalone IRT and IRT+BR-EKF filtering results of the mobile phone experimental procedure.

Method	$\mu_{e_{p}}$ [m]	RMSE [m]	$\mu_{\sigma_{\mathrm{xyz}}}$ [m]
IRT	74.483	77.498	157.035
IRT+BR-EKF	33.620	41.501	7.250

**Table 9:** Results from the Porto de Mós experimental procedure simulated with a ground perspective.

Method	$\mu_{e_{p}}$ [m]	RMSE [m]	$\mu_{\sigma_{\mathrm{xyz}}}$ [m]
IRT	134.377	140.2487	229.571
IRT+BR-EKF	73.923	94.686	4.055

**Table 10:** Results from the Porto de Mós experimental procedure simulated with an aerial perspective.

Method	$\mu_{e_{p}}$ [m]	RMSE [m]	$\mu_{\sigma_{\mathrm{xyz}}}$ [m]
IRT	47.120	47.605	43.560
IRT+BR-EKF	13.908	21.946	5.109

**Table 11:** Portuguese Air Force IRT and IRT+BR-EKF results.

Method	$\mu_{e_{p}}$ [m]	RMSE [m]	$\mu_{\sigma_{\mathrm{xyz}}}$ [m]
IRT	136.250	146.049	292.600
IRT+BR-EKF	198.434	157.056	24.7578

**Table 12:** UAVision footage results.

Frame	Landmarks	$\mu_{e_{p}}$ [m]	RMSE [m]	$\mu_{\sigma_{\mathrm{xyz}}}$ [m]
1	8	15.166	16.432	81.277
2	6	6.598	6.857	60.226
3	4	25.908	26.029	65.896

## Data Availability

Not applicable.

## Abbreviations

The following abbreviations are used in this manuscript:

**Abbreviation**	**Meaning**
BA	Bundle Adjustment
BO-CKF	Bearings-Only Cubature Kalman Filter
BO-EKF	Bearings-Only Extended Kalman Filter
BR-CKF	Bearings-Range Cubature Kalman Filter
BR-EKF	Bearings-Range Extended Kalman Filter
CKF	Cubature Kalman Filter
CRS	Coordinate Reference System
DEM	Digital Elevation Map
DOF	Degrees Of Freedom
ECEF	Earth-Centered Earth-Fixed
EKF	Extended Kalman Filter
EP	Extrinsic Parameters
GCP	Ground Control Points
GNSS	Global Navigation Satellite System
GPS	Global Positioning System
IMU	Inertial Measurement Unit
IP	Intrinsic Parameters
IPG	Iterative Photogrammetry
IRT	Iterative Ray-Tracing
MEMS	MicroElectroMechanical Systems
OCR	Optical Character Reader
QGIS	Quantum Geographic Information System
RMSE	Root Mean Squared Error
RT	Ray-Tracing
SfM	Structure from Motion
TIN	Triangular Irregular Network
UAV	Unmanned Aerial Vehicle
UKF	Unscented Kalman Filter
UT	Unscented Transform
WGS84	World Geodetic System 1984
